# Role of the Surgical Method in Development of Postoperative Cholangiocarcinoma in Todani Type IV Bile Duct Cysts

**DOI:** 10.1155/2015/417685

**Published:** 2015-07-09

**Authors:** Hong-tian Xia, Tao Yang, Bin Liang, Jian-ping Zeng, Jia-hong Dong

**Affiliations:** Hospital and Institute of Hepatobiliary Surgery, Chinese PLA General Hospital, Beijing 100853, China

## Abstract

*Background*. Our purpose was to investigate the association between the surgical approach for Todani type IV cysts and subsequent malignancy rate. *Methods*. The records of patients who received cyst excision from 1994 to 2013 were analyzed retrospectively for the following data: demographics, presenting symptoms, postoperative outcomes, malignant transformation, and follow-up reexaminations, including imaging, laboratory, and tumor marker tests. *Results*. Seven of the 196 patients initially treated at our hospital developed postoperative biliary malignancy, and the surgical approaches were extrahepatic bile duct cyst resection combined with hilar cholangioplasty and Roux-en-Y cystojejunostomy (*n* = 5), and intra- and extrahepatic bile duct cyst resection and Roux-en-Y hepaticojejunostomy (*n* = 2). The overall malignancy rate was 3.6% (7/196). Forty-eight patients initially treated at other hospitals developed malignancy postoperatively: 15 (31.2%) remained untreated and 33 (68.8%) had undergone incomplete resection procedures. Because Todani type IV cysts were seen in 268 patients, the postoperative malignancy rate of this group of patients was 12.3% (33/268). *Conclusions*. Radical resection of both intra- and extrahepatic cysts combined with hepatic resection and Roux-en-Y hepaticojejunostomy is associated with a reduced risk of subsequent cancer development. Procedures in which radical cyst excision is not performed are associated with a greater risk of subsequent malignancy.

## 1. Introduction

Congenital cystic dilatation of the bile duct, also known as choledochal cyst, is highly prevalent in Asia, especially in China [1, 2]. Choledochal cysts are currently one of the most common benign biliary tract diseases treated in our hepatobiliary surgery department, with over 50 cases treated annually. The most serious consequence of a choledochal cyst is the development of hepatobiliary cancer, and cholangiocarcinoma (CCC) is the most frequent histological type encountered [3, 4]. According to statistics from various centers in Asia, the cancer rate is approximately 8–15% and it can reach as high as 20% at specific centers [5–11]. Moreover, once CCC develops it is highly malignant with a poor outcome [2]. Therefore, the primary purpose of choledochal cyst surgery is to interrupt the natural course of CCC and prevent its development through early surgical removal of the cyst.

Most surgical interventions aimed at removing a choledochal cyst are successful in preventing the postoperative development of CCC [12]. However, the postoperative cancer rate is even higher in patients with Todani type IV choledochal cysts compared with patients who received no surgical intervention [13]. Analysis has shown that one of the causes of the high postoperative cancer rate is the type of surgical intervention employed [2, 3, 6, 14].

The aim of this study was to explore the relationship between the postoperative malignancy rate of Todani type IV bile duct cysts and the type of surgical method used to remove the cyst and to stress the importance of both intra- and extrahepatic cysts combined with hepatic resection and Roux-en-Y hepaticojejunostomy.

## 2. Materials and Methods

The ethics committee of our hospital approved this retrospective study. Every patient who received surgery signed an informed consent form detailing the surgery and procedures carried out during the operation. For patients who received surgery at other hospitals, ethics approval from other hospitals was not required as only the recorded information of past history of medical and surgical treatment was analyzed.

We reviewed all patients with a definite diagnosis of a choledochal cyst from January 1994 to June 2013. Data were collected using patients' medical records and operative reports. Patients' demographic information, presenting symptoms, surgical approaches for removal of choledochal cysts, postoperative outcomes, malignant transformation, and follow-up information were collected.

During postoperative follow-up, the patients were asked to come to our center for reexamination. For the first two years, reexamination occurred every three months and the examination items included computed tomography (CT) and magnetic resonance imaging (MRI) as well as biochemistry blood testing and tumor marker testing, such as CEA and CA19-9. Two years after surgery, for the patients from outside Beijing, reexamination occurred at the local hospitals instead of our center. If any abnormalities were observed in these patients, they would come to our center for reassessment. Our center keeps track of these nonlocal patients, and the reassessment items are consistent with those taken at the local hospitals. For the patients who remained asymptomatic for a certain period after surgery, reexamination could occur every six to twelve months.

The demographic and operative data were presented using descriptive analysis. For continuous variables, the mean ± standard deviation (SD) was calculated, and categorical data were presented as the number with or without percentage.

## 3. Results

### 3.1. Sample Development

**Figure 1:**
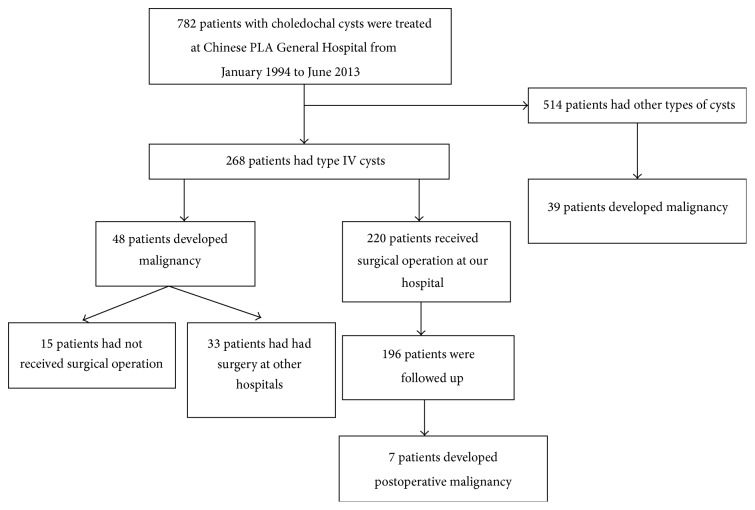
Flow diagram of patients' surgeries and outcomes. PLA: People's Liberation Army.

As shown in Figure 1, we identified 782 patients with choledochal cysts treated at the Hospital and Institute of Hepatobiliary Surgery of the Chinese PLA General Hospital. According to the classification proposed by Todani et al. [13], there are five types of choledochal cysts, and type IV cysts were seen in 268 patients (268/782, 34.3%). Overall, there were 87 patients who developed malignant transformation and the majority of these patients had an earlier diagnosis of Todani type IV cysts (48 of 87 patients; 55.2%). The median age of the 48 patients (12 males and 36 females) was 53.5 (range, 32–75) years. Among the 48 patients, 33 (33/48, 68.8%) had received surgery at a hospital other than ours, and 15 (15/48, 31.2%) remained untreated.

According to the data collected at our center over the past 20 years, the overall malignancy rate of Todani type IV bile duct cysts was 17.9% (48/268), and the postoperative malignancy rate was 12.3% (33/268).

### 3.2. Surgical Approaches and Postoperative Clinical Presentations

Table 1 summarizes the surgical approaches and postoperative clinical presentations of the 33 patients who received surgery at a hospital other than ours. Most patients (18/33, 54.5%) underwent simple internal drainage, that is, choledochoduodenostomy (CD) or choledochojejunostomy (CJ). In contrast, few patients (4/33, 12.1%) underwent external drainage via T-tube following bile duct exploration. There were 10 patients (10/33, 30.3%) who received extrahepatic cyst excision and Roux-en-Y hepaticojejunostomy and 1 patient (1/33, 3%) who received extrahepatic cyst excision and Roux-en-Y hepaticojejunostomy with partial liver resection. Cholangitis occurred in all patients (33/33, 100%). Other postoperative clinical features included abdominal pain in 27 (81.8%), fever in 25 (75.8%), jaundice in 22 (66.7%), cholangiolithiasis in 26 (78.8%), and biliary-enteric anastomotic stenosis in 23 (69.7%).

Of the 196 patients with follow-up data, 117 underwent extrahepatic bile duct cyst resection combined with hilar cholangioplasty and Roux-en-Y cystojejunostomy. The other 79 patients had intra- and extrahepatic bile duct cyst resection and Roux-en-Y hepaticojejunostomy with partial liver resection. The median follow-up duration of the 196 patients was 100 months (range, 18–226 months). As shown in Table 2, five of the 117 patients (4.3%) and two of the 79 patients (2.5%) developed postoperative malignancy, and the overall postoperative malignancy rate was 3.6% (7/196). They all had cholangitis with abdominal pain, fever, and jaundice as the presenting symptoms. These symptoms were present at follow-up time points and were not relieved until the malignancy was diagnosed.

## 4. Discussion

The results of this study indicate that cancer development after surgical treatment of Todani type IV bile duct cysts is related to the surgical approach. Radical resection of both intra- and extrahepatic cysts combined with hepatic resection and Roux-en-Y hepaticojejunostomy is associated with a reduced risk of subsequent cancer development. Procedures in which radical cyst excision is not performed are associated with a greater risk of subsequent cancer development.

To resolve the finding of a higher cancer rate after surgery in Todani type IV duct cysts, we analyzed the 33 patients who developed cancer after surgery at other hospitals. The results showed that simple internal drainage was the most commonly used surgical method, accounting for 54.6%, followed by extrahepatic bile duct cyst resection plus cholangioenterostomy, accounting for 30.3%, simple external drainage, accounting for 12.1%, and extrahepatic bile duct cyst excision and Roux-en-Y hepaticojejunostomy with partial hepatic resection, accounting for 3%. Of note is that the above 33 patients who developed cancer after surgery for Todani type IV cysts did not have radical removal of the bile duct cysts at the time of their initial surgery nor did they have adequate biliary drainage. We also found that all of the 33 patients had cholangitis, and this may be related to the subsequent malignancy because it is well known that recurrent cholangitis is a risk factor of cholangiocarcinoma [15]. These findings indicate that complete cyst excision is essential to prevent subsequent biliary malignancy from choledochal cysts.

Extrahepatic bile duct cyst resection plus Roux-en-Y hepaticojejunostomy has become a relatively common surgical treatment for Todani type IV bile duct cysts [16–22]. Surgical excision of cystic dilatation of the extrahepatic bile duct enables diversion of bile and pancreatic juice, which can to some extent slow the course of carcinogenesis within bile duct cysts. However, this surgical approach only removes the extrahepatic bile duct cysts and does not treat cystic dilatation of the intrahepatic bile ducts, thus leaving the problem of intrahepatic cholestasis (the pathological basis of carcinogenesis) unresolved. In addition, the presence of bilioenteric anastomosis allows intestinal reflux into the area of cystic dilatation within the intrahepatic bile ducts, causing regurgitation cholangitis. Long-term repeated episodes of cholangitis result in the formation of bile duct stones and stricture of the bilioenteric anastomotic stoma, contributing to carcinogenesis of bile duct cysts. Therefore, although it represents greater progress compared to internal and external drainage, extrahepatic bile duct cyst resection plus Roux-en-Y hepaticojejunostomy does not solve the problem of postoperative cancer development within Todani type IV bile duct cysts [23].

The ideal surgical method for treatment of Todani type IV bile duct cysts should (1) achieve diversion of the bile and pancreatic juice and (2) enable adequate biliary drainage. Based on these two criteria, radical resection of the involved bile ducts and restoration of normal physiological function of the bile ducts are necessary. Thus, radical resection of intra- and extrahepatic bile duct cysts combined with partial hepatic resection and Roux-en-Y hepaticojejunostomy represents the ideal surgical method for the treatment of Todani type IV bile duct cysts.

If the intrahepatic bile duct lesions are confined to a segment or a lobe of the liver, radical resection of intra- and extrahepatic bile duct cysts combined with partial hepatic resection and Roux-en-Y hepaticojejunostomy can achieve curative resection of the bile duct lesions. Patients who received radical resection of Todani type IV bile duct cysts had a zero rate of cancer (data not shown). However, in actual clinical practice, not all Todani type IV patients have bile duct lesions confined to a hepatic segment or lobe. Rather, a certain proportion of patients have widely distributed intrahepatic bile duct lesions, and because of the limitations of liver resection a partial hepatectomy cannot achieve radical resection of all bile duct lesions. The only surgical method that satisfies all the above conditions is liver transplantation, which is unrealistic for most Todani type IV cyst patients. Therefore, there are only two surgical options: extrahepatic bile duct cyst excision plus hilar cholangioplasty and Roux-en-Y hepaticojejunostomy or partial hepatectomy plus intra- and extrahepatic bile duct cyst resection and Roux-en-Y hepaticojejunostomy. Both can achieve diversion of bile and pancreatic juice and improve biliary drainage.

Our data showed there were seven cases of postoperative cancer in Todani type IV bile duct cysts patients, with an overall cancer rate of 3.6%. Thus, although a certain percentage of patients developed postoperative cancer after treatment using these two surgical methods, the cancer rate was greatly reduced. Of particular note, due to widely distributed intrahepatic bile duct lesions, complete resection was not achieved in these seven patients although the surgical techniques were the same as those in the remaining 189 patients. This is the fundamental reason for cancer developing following surgery for Todani type IV bile duct cysts and confirms the conclusion that radical resection of intra- and extrahepatic bile duct cysts combined with partial hepatic resection and Roux-en-Y hepaticojejunostomy represents the ideal surgical method for surgical treatment of Todani type IV cysts.

This study has several limitations including its retrospective nature, which precludes the data collection of the total number of patients receiving operation at other hospitals. In addition, the follow-up length of several patients was relatively short, which may have affected the results. Future research findings with longer follow-up times are, therefore, needed to confirm our findings.

## 5. Conclusions

In summary, postoperative malignancy in Todani type IV bile duct cysts appears closely related to the surgical approach used. Radical resection of both intra-and extrahepatic cysts, combined with hepatic resection and Roux-en-Y hepaticojejunostomy, is associated with a reduced rate of subsequent malignancy and thus may constitute the ideal surgical method for the treatment of Todani type IV bile duct cysts. When radical resection of the cysts cannot be achieved, the rate of cancer development after surgery can be reduced as long as adequate bile drainage is provided during surgery.

## Figures and Tables

**Table 1:** Surgical approaches and postoperative clinical presentations of 33 patients treated at other hospitals who developed postoperative malignancy.

Clinical presentation	Surgical approach					
	CD (*n* = 7)	CJ (*n* = 11)	Extrahepatic cyst excision and Roux-en-Y HJ (*n* = 10)	Extrahepatic cyst excision and Roux-en-Y HJ with partial liver resection (*n* = 1)	T-tube drainage after bile duct exploration (*n* = 4)	Total number (%)
Cholangitis	7	11	10	1	4	33 (100)
Abdominal pain	5	7	10	1	4	27 (81.8)
Fever	6	9	6	1	3	25 (75.8)
Jaundice	4	10	5	1	2	22 (66.7)
Cholangiolithiasis	5	8	8	1	4	26 (78.8)
Biliary-enteric anastomotic stenosis	3	9	10	1	0	23 (69.7)

CD: choledochoduodenostomy; CJ: choledochojejunostomy; HJ: hepaticojejunostomy.

**Table 2:** Demographic and clinical data of seven patients treated at our hospital who developed postoperative malignancy.

Previous surgery	Case number	Age (years)	Gender	Follow-up (months)	Postoperative clinical presentation	Interval between operation and malignancy (months)
Extrahepatic bile duct resection combined with hilar cholangioplasty and Roux-en-Y cystojejunostomy	1	36	Female	109	Abdominal pain, fever	42
	2	52	Male	65	Fever	76
	3	57	Female	178	Abdominal pain, fever, and jaundice	98
	4	65	Female	92	Abdominal pain, jaundice	152
	5	68	Female	198	Abdominal pain, fever	190

Intra- and extrahepatic bile duct cyst resection and Roux-en-Y hepaticojejunostomy	6	41	Female	97	Abdominal pain, fever, and jaundice	82
	7	62	Male	180	Abdominal pain, fever	162
